# Effects of Salts and Other Contaminants on Ciprofloxacin Removal Efficiency of Green Synthesized Copper Nanoparticles

**DOI:** 10.3390/vetsci11040179

**Published:** 2024-04-16

**Authors:** Tanongsak Sassa-deepaeng, Nattakanwadee Khumpirapang, Wachira Yodthong, Yin Yin Myat, Songyot Anuchapreeda, Siriporn Okonogi

**Affiliations:** 1 Agricultural Biochemistry Research Unit, Faculty of Sciences and Agricultural Technology, Rajamangala University of Technology Lanna Lampang, Lampang 52000, Thailand; tanongsaks@rmutl.ac.th; 2 Department of Pharmaceutical Chemistry and Pharmacognosy, Faculty of Pharmaceutical Sciences, Naresuan University, Phitsanulok 65000, Thailand; nattakanwadeek@nu.ac.th; 3 Lampang Inland Fisheries Research and Development Center, Lampang 52000, Thailand; wachira.yodthong@gmail.com; 4 Center of Excellence in Pharmaceutical Nanotechnology, Faculty of Pharmacy, Chiang Mai University, Chiang Mai 50200, Thailand; yinyinmyat87@gmail.com (Y.Y.M.); songyot.anuch@cmu.ac.th (S.A.); 5 Department of Medical Technology, Faculty of Associated Medical Sciences, Chiang Mai University, Chiang Mai 50200, Thailand; 6 Department of Pharmaceutical Sciences, Faculty of Pharmacy, Chiang Mai University, Chiang Mai 50200, Thailand

**Keywords:** *Garcinia mangostana*, ciprofloxacin, antibiotic removal, plant extract, copper nanoparticles, fish wastewater, aquaculture

## Abstract

**Simple Summary:**

In this study, copper nanoparticles synthesized using *Garcinia mangostana* extract (GME-CuNPs) were evaluated for their elimination efficiency of ciprofloxacin (CIP). Our findings demonstrate that their CIP removal efficiency (CRE) is affected by salts and some contaminants in fish wastewater but not by certain phenolic compounds or ammonia. The electrostatic attraction between negatively charged GME-CuNPs and cationic CIP species controls the CRE. Adjustment of the pH to 6–7 in fish wastewater is crucial for optimal CRE. Overall, GME-CuNPs demonstrate effective elimination of CIP from fish wastewater. This suggests the potential of GME-CuNPs as a promising tool for environmental antibiotic elimination.

**Abstract:**

Ciprofloxacin (CIP), a broad-spectrum fluoroquinolone antibiotic, is commonly used in aquaculture to prevent and treat bacterial infections in aquatic animals. For this reason, aquatic environments contain CIP and its derivatives, which lead to the development of drug-resistant bacteria. In the present study, copper nanoparticles were prepared using *Garcinia mangostana* extract (GME-CuNPs) as a reducing agent and evaluated for their CIP removal efficiency (CRE). The results demonstrate that within 20 min, GME-CuNPs at 25 mM possess a CRE of 92.02 ± 0.09% from CIP-containing aqueous media with pH 6–7. The CRE is influenced by both monovalent and divalent salts. A high salt concentration significantly reduces the CRE. Contaminants in fish wastewater can reduce the CRE, but phenolics, flavonoids, tannins, and ammonia do not affect the CRE. Our results reveal that the CRE is controlled by electrostatic attraction between the negatively charged GME-CuNPs and the cationic species of CIP. The CRE is reduced by wastewater with a pH higher than 8.0, in which the CIP molecules have a negative charge, resulting in a repulsive force due to the negative charge of GME-CuNPs. In fish wastewater with a pH lower than 7.0, GME-CuNPs show the potential to achieve a CRE above 80%. Therefore, pH adjustment to a range of 6–7 in fish wastewater before treatment is deemed imperative. It is concluded that the newly developed GME-CuNPs possess excellent activity in CIP elimination from actual fish wastewater samples. Our findings suggest that GME-CuNPs can be a promising tool to effectively eliminate antibiotics from the environment.

## 1. Introduction

**Figure 1:**
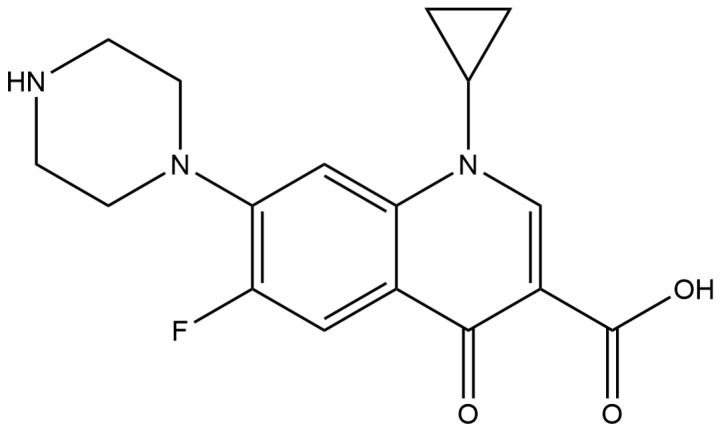
Chemical structure of CIP.

Ciprofloxacin (CIP) is a broad-spectrum antibiotic in the fluoroquinolone group that possesses effective antibacterial activity against a variety of Gram-positive and Gram-negative bacteria. According to the chemical structure of CIP, as shown in Figure 1, CIP molecules contain an aromatic ring, a quinolone ring, a carboxyl group (pKa = 6.1), an a-keto moiety, a protonated secondary amino group (pKa = 8.6), and a halogen. CIP effectively regulates bacterial DNA supercoiling, a procedure necessary for DNA replication, recombination, and repair, by binding to and inhibiting DNA gyrase enzymes [1]. Thus, CIP is recommended for treating tuberculosis, campylobacter infections, and salmonellosis in humans, as well as treating a number of pathogenic bacteria associated with diseases of aquatic animals, both marine and freshwater [2,3,4,5].

For fish culturing in both farms and aquaria, CIP is commonly used to prevent and treat infectious diseases in both food and ornamental fish. However, there are increasing reports of CIP resistance in a number of pathogenic bacteria. These include *Pseudomonas aeruginosa*, *Bacillus anthracis*, *Neisseria gonorrhoeae, Enterococci*, *Escherichia coli*, *Edwardsiella tarda*, *Klebsiella pneumoniae*, and *Staphylococcus aureus*. This resistance is attributed to the presence of residual CIP in wastewater that is discharged into the environment because residual drugs activate bacterial antibiotic-resistance genes [5,6]. Many strategies have been considered to remove CIP in aquaria when the treatment of a bacterial infection is completed. One of the effective methods of CIP removal is the use of metal nanoparticles. Copper nanoparticles (CuNPs) have been used to remove pharmaceutical pollutants, such as ibuprofen, naproxen, and diclofenac, out of aquatic systems [7]. Our previous study showed that *Garcinia mangosteen* leaf extract possessed the highest reducing power among other potential plants and influencing the redox reactions involved in the formulation of metal nanoparticles. CIP added to the pure water system could be successfully eliminated using CuNPs synthesized from *G. mangosteen* extract [8]. However, wastewater from fish contains various substances, such as salts and other contaminants secreted by fish. This may affect the removal efficacy of CIP from the system. In addition, different fish species may secrete different substances that can lower CIP removal rates. Therefore, in this study, various wastewater systems generated by different CIP-treated fish species, such as *Betta splendens*, *Carassius auratus*, *Cyprinus carpio*, *Oreochromis niloticus*, *Poecilia reticulate*, and *Systomus rubripinnis*, were investigated.

*B. splendens*, commonly called Siamese fighting fish, or simply “betta”, is a popular aquarium ornamental fish in global trade because of their beautiful morphology with variations in color [9]. However, they are often infected by several pathogenic bacteria, such as *Aquitalea pelogenes*, *Acinetobacter pittii*, *Bacillus cereus*, *Pseudomonas mosselii*, *Acinetobacter baylyi*, *Klebsiella variicola*, *Aeromonas veronii*, and *Mycobacterium marinum* [10,11]. Thus, many fluoroquinolones are used to control infectious diseases in this type of fish [12]. *C. auratus* is a freshwater ornamental fish that is popularly cultured for decorative purposes and its important economic value. Many pathogenic bacteria, such as *Aeromonas hydrophila*, *Rahnella aquatilis*, and *Edwardsiella ictaluri* [13,14], have been reported to cause severe infections, leading to enormous economic losses in the *C. auratus* breeding industry. To avoid devastation in farming, fluoroquinolones, including CIP, are widely used to prevent and treat bacterial infections in these fish [15]. *C. carpio*, or common carp, is in the family Cyprinidae, the largest family of freshwater fish. This is a very important aquatic species in Asia and some countries in Europe [16]. Common carp are extremely tolerant of a wide range of environmental conditions, including extremely low dissolved oxygen and high turbidity. However, they have been reported to be infected with *Aeromonas salmonicida*, *Vibrio salmonicida*, *Vibrio anguillarum*, and *Yersinia rucheri* but could be recovered by treatment with CIP [17]. *O. niloticus*, or Nile tilapia, was one of the first species bred by humans [18]. The bacterial infections of Nile tilapia have been reported to be successively treated with fluoroquinolones; however, some reports have indicated that the antibiotic residues, specifically CIP, were detected in the muscles and liver of these economically important fish [3,19]. *P. reticulate* is a popular ornamental fish that has also employed CIP for controlling bacterial diseases, especially those infected with *Aeromonas* spp., which brings huge losses to the ornamental fish industry [20]. *S. rubripinnis* is a freshwater fish belonging to the family Cyprinidae [21]. They can be commonly found in rivers or canals with flowing streams in Asian countries, especially Thailand and Indonesia. In Thailand, they are captured from the wild for the ornamental fish trade. They are always treated with CIP to manage bacterial infections. This research aimed to collect wastewater from these fish to examine the efficiency of synthesized CuNPs in removing CIP under various conditions.

## 2. Materials and Methods

### 2.1. Chemical Materials

Various chemical reagents were sourced from suppliers across different regions. Ferric chloride and sodium nitrite were acquired from Ajax Finechem (New South Wales, Australia). Gallic acid and CIP hydrochloride were purchased from Bio Basic Inc. in Markham, ON, Canada. Epigallocatechin gallate and quercetin were procured from Sigma–Aldrich (Steinheim, Germany). Copper sulfate pentahydrate and hydrochloric acid were purchased from QRëC (Auckland, New Zealand). Chloroform and acetic acid were from JT Baker (Phillipsburg, NJ, USA). Folin–Ciocalteu reagent was purchased from Carlo Erba (Barcelona Spain). Ammonium chloride, magnesium chloride hexahydrate, manganese sulfate, phenol detached crystals, and sodium hypochlorite were acquired from Loba Chemie (Mumbai, India). Aluminum chloride hexahydrate, bromophenol blue, sodium acetate, and calcium carbonate were acquired from KemAu (Sydney, Australia). Vanillin was purchased from Merck (Darmstadt, Germany). We obtained sodium hydroxide and sodium chloride from RCI Labscan located in Thailand. Other chemicals and solvents were of the highest grade available.

### 2.2. Plant and Extract Preparation

*Garcinia mangostana* was harvested in northern Thailand, from July to August 2022. Botanical identification and authentication of plant material were performed by an herbalist affiliated with Rajamangala University of Technology Lanna, where reference specimens are kept. Fresh leaves were carefully separated from the stems and meticulously cleansed with tap water followed by deionized water. Subsequently, the leaf samples were dried at 50 °C in a hot air oven until a consistent weight was reached. After drying, an electric blender was used to finely grind the dried leaves. The obtained plant material was sieved through a 20-mesh screen before extract preparation. An aqueous extract of *G. mangostana* (GME) was prepared as follows: a mixture consisting of 1 g of dried plant powder and 100 mL of distilled water was heated at 85 °C for a period of 30 min, after which it was allowed to cool to room temperature. The cold mixture was then passed through a 0.22-micron nylon syringe filter (Monotaro, Thailand). The resulting filtrate served as a GME material for CuNP synthesis.

### 2.3. Chemical Analysis of GME

The GME was analyzed for its chemical composition using liquid chromatography with an electrospray ionization quadrupole time-of-flight mass spectrometer (LC-ESI-QTOF) technique. An Agilent 1260 Infinity series high-performance liquid chromatography system (Agilent Technologies, Waldbronn, Germany) was used, coupled with a 6540 ultra-high-definition accurate mass spectrometer (Agilent Technologies, Singapore). The GME was separated on a Luna C18(2) (150 mm × 4.6 mm, 5 μm) (Phenomenex, Torrance, CA, USA) at a flow rate of 0.5 mL/min, with an injection volume of 10 μL. The column compartment was kept at a temperature of 35 °C. The mobile phase consisted of a combination of A (0.1% formic acid in water type I, *v*/*v*) and B (0.1% formic acid in acetonitrile, *v*/*v*). The gradient elution was established as follows: 0–30 min, 95:5 to 5:95 (A/B *v*/*v*), and held for 10 min. Afterward, the re-equilibration was 5 min.

Additionally, mass spectrometry analysis was conducted using an Agilent dual nebulizer equipped with an electrospray ionization (ESI) source, operating in both the negative and positive ionization modes to acquire comprehensive data. The following parameters were employed: a drying gas (N_2_) flow rate of 10.0 L/min; drying gas temperature of 350 °C; nebulizer pressure of 30 psig; capillary voltage of 3.5 kV; skimmer voltage of 65 V; and Octopole RF peak of 750 V. The mass-to-charge ratio (*m*/*z*) range was set from 100 to 1000 Da. Fragmentation patterns were generated with collision energies set to 10, 20, and 40 V. The chemical constituents were identified by their accurate *m*/*z* and fragmentation patterns; the data were compared with published data from a library search, i.e., METLIN Metabolite Personal Compound Database (PCD, Agilent Technologies) and Human Metabolome (https://hmdb.ca/, accessed on 16 January 2024).

### 2.4. Green Synthesis of CuNPs

The synthesis of CuNPs in the present study was performed using copper sulfate as the precursor and GME as the reducing agent. The process of synthesis was in accordance with a method previously described [8]. Briefly, 200 mL of GME was diluted with 300 mL of deionized water and mixed with 500 mL of 50 mM copper sulfate in deionized water. For the reduction of metal salt, the mixture was refluxed in a dry incubator at 80 °C for a period of 30 min. The dispersion color transitioned from clear to yellow and then became light brown. This functioned as an observable signifier of the full development of the intended metal nanoparticles (GME-CuNPs), which possessed physicochemical characteristics as previously described [8]. The obtained GME-CuNP dispersion was cooled to an ambient temperature before further use. The GME-CuNP concentration in the obtained colloidal dispersions was calculated using the method previously described [22]. Principally, the number of metal atoms in the prepared metal nanoparticles was obtained from the amount of initial precursor used for synthesis. For example, the amount of copper sulfate, which was the precursor for synthesizing GME-CuNPs, was assumed to be equal to the copper atoms in the final colloidal suspension. Further diluted concentrations were assumed using a 2-fold dilution technique.

### 2.5. CIP Removal Efficiency (CRE) of GME-CuNPs

A variety of scientific theories and techniques, including mass spectrometry, spectrophotometry, and high-performance liquid chromatography, have been used to determine the CIP for the calculation of the CIP removal efficiency (CRE). In the current study, an aqueous solution of 25 mg% CIP was prepared by dissolving 25 mg of CIP powder in 100 mL of DI water, and then mixing it with GME-CuNP colloidal dispersions of various concentrations, and the mixtures were left at room temperature for 20 min. The samples were then centrifuged at 500 rpm for a period of 10 min. A mixture consisting of 250 µL of each supernatant, 375 µL of 0.1% *v*/*v* bromophenol blue, and 100 µL of 20 mM acetate buffer (pH 4.1) was prepared. Subsequently, 2.5 mL of chloroform was added to the mixture, and it was rapidly shaken for 30 s. After phase separation occurred, the yellow layer, containing a yellow compound resulting from an ion-pair complex formation between the CIP and bromothymol blue, was separated and measured using spectrophotometry at 416 nm [23]. A calibration curve was constructed using diluted CIP at various concentrations (R^2^ = 0.999). The system without GME-CuNPs served as a control. The CRE was calculated using Equation (1).

CRE (%) = [(*Ac* − *As*)/*Ac*] × 100, (1)

where *Ac* is the absorbance of the control, and *As* is the absorbance of the sample.

### 2.6. Effects of Salts on CRE

To investigate the effects of salts on the CRE of GME-CuNPs, various concentrations of monovalent and divalent salts were added to the 25 mg% CIP solution. Sodium chloride was used as a representative of monovalent salts. Divalent salts, such as calcium chloride and magnesium chloride, were used as they are the most common source of water hardness. The colloidal dispersion of GME-CuNPs was mixed with a freshly prepared salt solution containing CIP, resulting in a GME-CuNP concentration of 25 mM for monovalent salt, and within the range of 0.2–100 mM for divalent salts. These mixtures were allowed to stand at room temperature for 20 min, and then centrifuged for 10 min at 500 rpm. A 250 µL portion of the supernatant was blended with 100 µL of 20 mM acetate buffer (pH 4.1) and 375 µL of 0.1% *v*/*v* bromophenol blue. After that, 2.5 mL of chloroform was added to the mixture, and it was shaken vigorously for 30 s to facilitate phase separation between the chloroform and the aqueous layer. The absorbance of the yellow layer, which originated from ion-pair complexes formed between the CIP and bromothymol blue, was measured at 416 nm. The system without GME-CuNPs was employed as a control. CRE was calculated using Equation (1).

### 2.7. Effects of Fish Wastewater on CRE

Fish wastewater samples were collected from public aquariums, an ornamental fish farm, and an aquaculture practice farm for students at the Department of Animal Science and Fishery, Faculty of Science and Agricultural Technology, Rajamangala University of Technology Lanna, Thailand. In addition, all wastewater samples were sourced from aquaculture systems housing *Betta splendens*, *Carassius auratus*, *Cyprinus carpio*, *Oreochromis niloticus*, *Poecilia reticulate*, and *Systomus rubripinnis*. The collected samples were pre-treated by filtration to remove any pieces of debris. The pH levels of these freshly prepared samples were determined using a pH meter (CyberScan 500, Singapore). The ammonia content was determined using methods previously described [24,25]. The total ammonia content was expressed as the ammonium chloride (NH_4_Cl) equivalent. Furthermore, the total phenolic, flavonoid, and tannin contents of the wastewater samples were also determined using a method previously described [8]. The total phenolic content and total flavonoid content were expressed as the gallic acid equivalent (GAE) and quercetin equivalent (QE), while the total tannin content was expressed as the epigallocatechin 3-gallate (EGCG) equivalent. In brief, the fish wastewater samples were mixed with the respective reagents according to each protocol. Subsequently, the final products were measured for absorbance using a microplate reader.

To investigate the CRE of GME-CuNPs in each fish wastewater sample, CIP was added to the wastewater samples to obtain a CIP concentration of 25 mg%. Subsequently, GME-CuNP dispersion was added to the CIP-containing fish wastewater samples to reach a final GME-CuNP concentration of 25 mM. The resulting combinations were centrifuged for 10 min at 500 rpm. Next, 250 µL of the supernatant was mixed with 375 µL of 0.1% *v*/*v* bromophenol blue and 100 µL of a 20 mM acetate buffer (pH 4.1). Then, t 2.5 mL of chloroform was added to the mixture, and it was vigorously shaken for 30 s to facilitate phase separation between the chloroform and the aqueous layer. The absorbance of the yellow layer was measured at 416 nm. Fish wastewater without GME-CuNPs served as a control. The CRE was calculated using Equation (1).

### 2.8. Statistical Analysis

The data are presented as the mean ± standard error of the mean (S.E.M.) from three independent experiments. The normality of the data was checked using the Kolmogorov–Smirnov’s test, skewness, and kurtosis. The variance homogeneity was determined using Levene’s test. ANOVA, followed by Tukey’s post hoc test, was used to analyze the data of the CRE of the GME-CuNPs. Probability values of less than 0.05 (*p* < 0.05) were considered statistically significant.

## 3. Results

### 3.1. Chemical Constituents of GME

**Figure 2:**
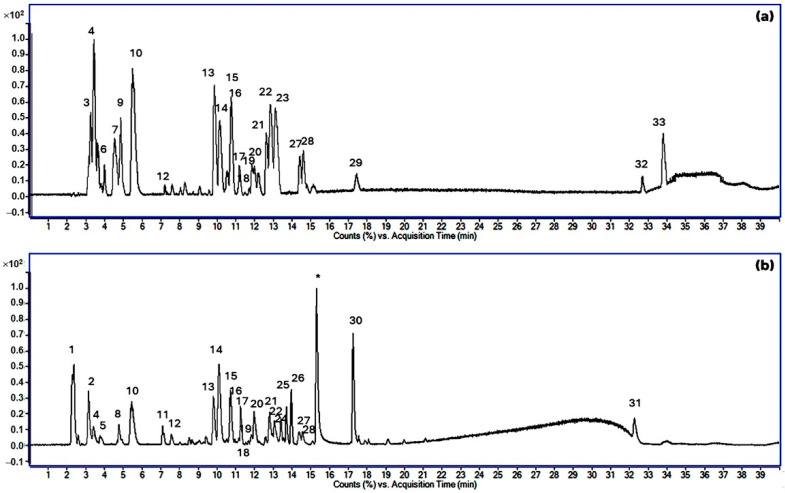
The total ion chromatograms (TIC) of the GAE in the negative mode (**a**) and positive mode (**b**). The peak numbers of compounds in (**a**,**b**) correspond to those in Table 1. (* is the contamination peak).

In an LC-ESI-QTOF experiment in which the negative and positive ionization modes were used for the analysis of GME, numerous polyphenolics, phenolics, flavonoids, tannins, xanthones, and carboxylic acid derivative compounds were detected, as shown in Figure 2 and Table 1. In the negative ionization mode, quinic acid exhibited the highest signal intensity, followed by ascorbic acid-6-acetate, caffeoylquinic acid, and catechin, respectively. In the positive ionization mode, caryatin glucoside, procyanidin B2, and catechin showed the highest signal intensities.

### 3.2. Green Synthesis of GME-CuNPs

*G. mangostana* was proven to be a suitable candidate for the synthesis of metal nanoparticles and is an important component in achieving the objective of sustainable green synthesis. Using the prepared GME in the current study, it was found that GME-CuNPs could be successfully formed. With a narrow size distribution, expressed as a polydispersity index (PdI) of 0.28 ± 0.01 with an average particle diameter of 158 ± 4.04 nm, the produced metal nanoparticles displayed nanoscale features. Furthermore, the GME-CuNPs revealed a negative surface charge with a zeta potential of −4.95 ± 1.27 mV.

### 3.3. Effects of Sodium Chloride on CRE of GME-CuNPs

**Figure 3:**
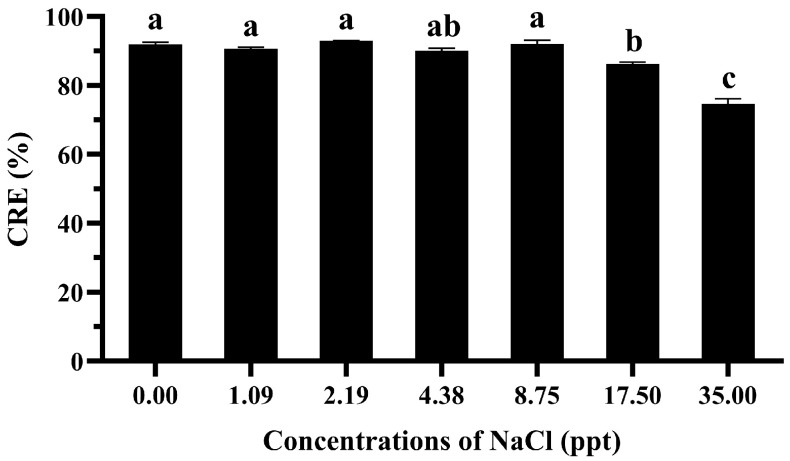
Effect of NaCl concentrations on CRE of 25 mM GME-CuNPs in a 25 mg% CIP water solution. Data are presented as means ± S.E.M. Different lowercase letters indicate significant differences among NaCl concentrations, based on one-way ANOVA and Tukey’s test (*p* < 0.05).

The results indicate that the maximum CRE achieved by the GME-CuNPs was 92.98 ± 0.08% when employing a sodium chloride concentration of 2.19 ppt, as illustrated in Figure 3. However, the statistical analysis indicated that there was no significant difference in the CRE at salt concentrations ranging from 0.00 to 8.75 ppt. A slightly significant decrease in the CRE was observed, declining from 92.07% ± 1.07% to 86.17% ± 1.12%, when the salt concentration was increased from 8.75 to 17.50 ppt. Notably, the most significant reduction in the CRE was observed at a salt concentration of 35.00 ppt, which resulted in a CRE value of 74.63% ± 1.52%.

### 3.4. Effects of Divalent Salts on CRE of GME-CuNPs

**Figure 4:**
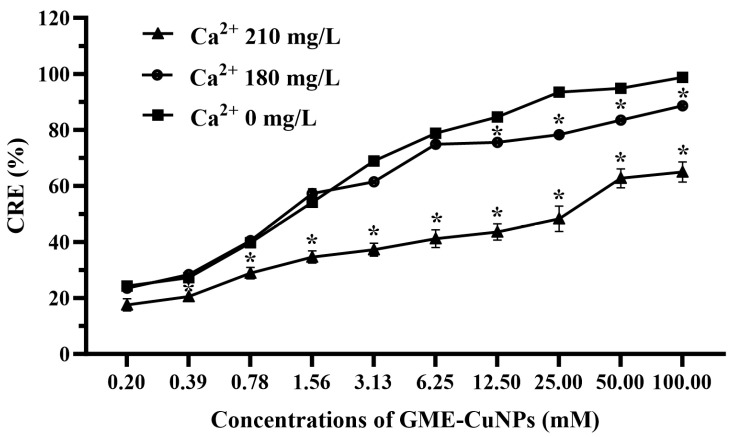
Effect of calcium salt on CRE of GME-CuNPs at various concentrations. Asterisks (*) indicate significant differences compared to group without calcium ions (*p* < 0.05).

In this experiment, the effects of two divalent salts of calcium and magnesium were investigated. In general, the classification of water containing calcium ion is as follows: 0 to 60 mg/L of calcium carbonate is classified as soft; 61 to 120 mg/L as moderately hard; 121 to 180 mg/L as hard; and more than 180 mg/L as very hard [26]. In most aquaculture, water that is hard to very hard is often used. Therefore, in this experiment, aqueous solutions of calcium chloride at 180 and 210 mg/L were used. The results are shown in Figure 4.

**Figure 5:**
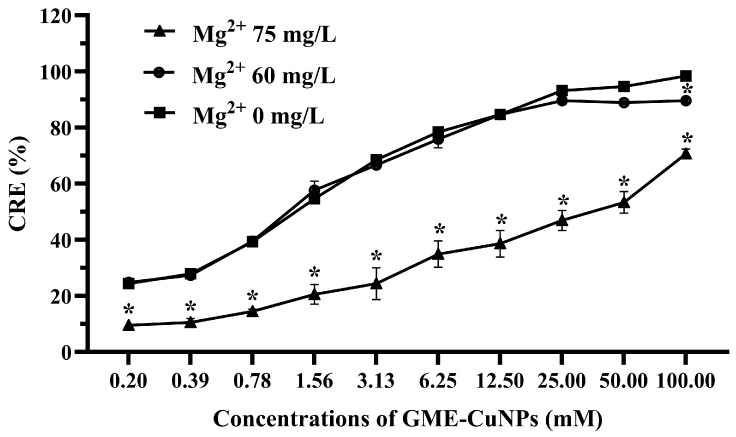
Effect of magnesium salt on CRE of GME-CuNPs at various concentrations. Asterisks (*) indicate significant differences compared to group without magnesium ions (*p* < 0.05).

From the results, it was found that the effect of calcium salt at 180 mg/L on the CRE of the GME-CuNPs depended on the concentration of GME-CuNPs. At low concentrations, the CRE of the GME-CuNPs of this system was the same as that without calcium ions. However, at GME-CuNP concentrations of 12.50 mM or higher, the CRE of this system was significantly reduced. When increasing the calcium salt concentration to 210 mg/L, the CRE was significantly reduced for all concentrations of GME-CuNPs. These results indicate that calcium ions play an important role in the CRE of GME-CuNPs. For magnesium salt, in the present study, magnesium chloride solutions at concentrations of 0, 60, and 75 mg/L were studied, and the results are shown in Figure 5. It is clearly seen that water containing Mg^+2^ at 75 mg/L significantly affected the CRE of the GME-CuNPs at all tested concentrations.

### 3.5. CRE of GME-CuNPs in Fish Aquaria

**Figure 6:**
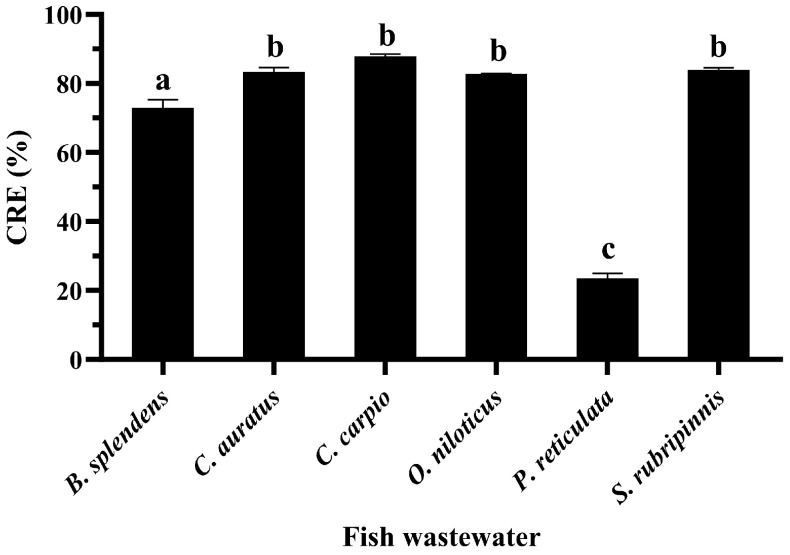
CRE of 25 mM GME-CuNPs in fish wastewater samples containing 25 mg% CIP and control water (without fish) containing 25 mg% CIP. Data are presented as means ± S.E.M. Different lowercase letters indicate significant differences among fish wastewater samples, based on one-way ANOVA and Tukey’s test (*p* < 0.05).

**Figure 7:**
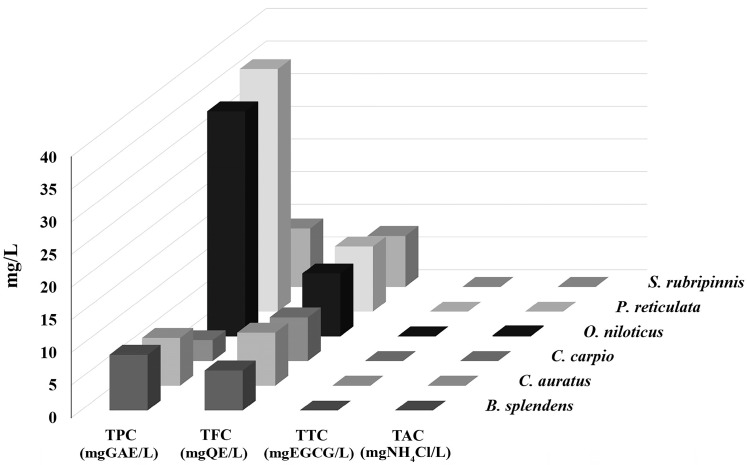
Total phenolic content (TPC), total flavonoid content (TFC), total tannin content (TTC), and total ammonia content (TAC) in wastewaters of different fish species.

It was found that the CRE of GME-CuNPs from the fish wastewater of all fish species was significantly lower than that from the CIP solution in pure water. However, the CRE reduction in the wastewater of each fish species was different. The CRE of the fish wastewater samples from *B. splendens*, *C. auratus*, *C. carpio*, *O. niloticus*, and *S. rubripinnis* ranged from above 73 to nearly 90%, as shown in Figure 6. Meanwhile, the CRE processed in the *P. reticulate* wastewater decreased significantly to 23.55 ± 1.37%. To investigate the factors that might affect the CRE of this wastewater, the total phenolics, flavonoids, tannins, and ammonia, which contribute to the antioxidant properties, were examined in all samples. The results are shown in Figure 7. It can clearly be seen that total tannin content and total ammonia content of all samples were found to be negligible and not significantly different.

**Figure 8:**
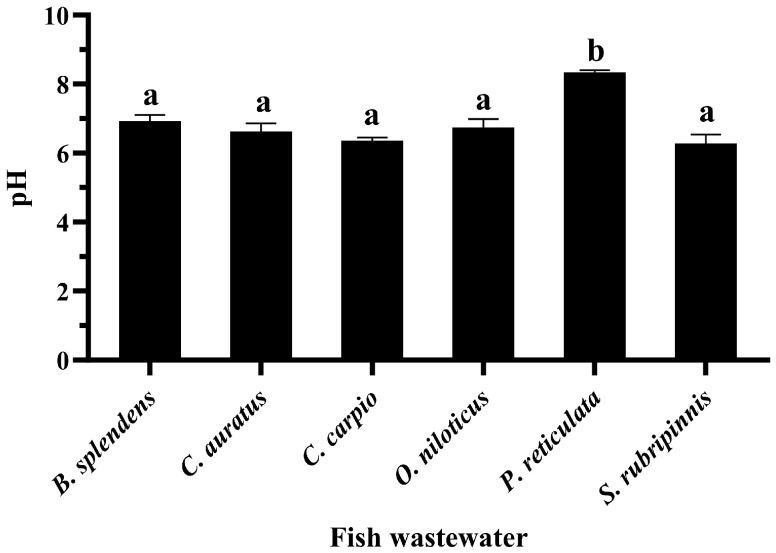
pH values of fish wastewater. Data are presented as means ± S.E.M. Different lowercase letters indicate significant differences among fish wastewater samples, based on one-way ANOVA and Tukey’s test (*p* < 0.05).

Further studies of the pH measurement in three independent experiments yielded interesting results. It was found that the pH value of the *P. reticulata* wastewater was greater than 8, while the pH value of the wastewater from other fish species was less than 7, as shown in Figure 8.

## 4. Discussion

CIP is one of the most promising drugs for use in aquaculture to prevent and treat bacterial infections in aquatic animals. However, the resistance of bacteria to CIP has become a big problem. Therefore, the effective elimination of CIP is required. Various scientific principles, including biological treatments and sorption technologies, have been used. Activated sludge systems are examples of biological treatment processes that can facilitate the biodegradation of CIP by microbial communities [27]. CIP removal can occur through sorption processes onto solid surfaces, such as activated carbon, clays, or other adsorbents. The efficiency of removal depends on factors, such as the surface area and porosity of the adsorbent, as well as the physicochemical properties of CIP and the water matrix [28]. Metal nanoparticles, such as copper, iron, and manganese [29,30], have emerged as convenient and promising options for the elimination of CIP via adsorption mechanisms [31,32,33]. Various techniques have been developed for the synthesis of these metal nanoparticles, including laser ablation, electric arc discharge, microwave-assisted processes, co-precipitation, vacuum vapor deposition, high-energy irradiation, lithography, and methods using plant extracts as reducing agents, also known as the green synthesis method [34,35,36]. Among these techniques, green synthesis has garnered increasing attention due to its cost-effectiveness and environmental friendliness [37]. Furthermore, green synthesis necessitates only basic instruments and plant materials, and it is considered rapid and non-toxic in comparison to other techniques [38]. However, a critical requirement for green synthesis is the presence of potential plant extract with high antioxidant activity to facilitate the reduction of metal ions. *G. mangostana*, belonging to the Clusiaceae family, is native to tropical regions in Southeast Asia, southern Africa, America, and Australia [39]. Garcinia species have been recognized as a rich source of phenolic metabolites with robust antioxidant and high reducing properties, making them valuable contributors as reducing agents in metal nanoparticle synthesis [40,41]. All parts of Garcinia species, including the pericarps, leaves, bark, stems, fruits, seeds, and flowers, have abundant phenolic compounds, making them particularly promising as potential catalysts in comparison to other plant sources [42,43]. The results of the current study that GME possesses numerous phenolic compounds, notably procyanidin B2, a potent antioxidant agent. Flavonoids, such as rutin, astrilbin, and caryatin glucoside; tannins, including catechin, caffeoylquinic acid, and epicatechin-(4beta->8)-gallocatechin; and xanthones, such as gamma-mangostin and its derivatives, and carboxylic acid derivative compounds like quinic acid are also presented in GME. These results confirm that *G. mangostana* has high potential as a plant for use as a reducing agent in the synthesis of metal nanoparticles, like copper nanoparticles.

Copper nanoparticles have a high surface area, which allows them to adsorb antibiotic molecules onto their surfaces. This adsorption process occurs through weak interactions, such as electrostatic forces, hydrogen bonding, or van der Waals forces, or by undergoing redox reactions with antibiotics. It has been reported that *G. mangostana* contains high TPC, TFC, and TTC [8,44]. These polyphenolic and flavonoid molecules can be adsorbed on the nanoparticle surface [45] and increase the negative particle surface charge [27]. Therefore, GME-CuNPs have a high ability to react with positively charged functional groups in CIP molecules, leading to changes in the chemical structure, biological activity, and stability of the drug.

Salt baths are often used to prevent fungal infections in fish, recover blood salts after stress, mitigate harmful environmental conditions, and restore fish osmoregulation. Adding a small amount of monovalent salt, such as sodium chloride, into the water is a traditional and effective way to reduce stress and treat sick fish [46,47]. In addition, the average sodium chloride concentration for sea water salinity is approximately 35 g/L or 35 ppt [48]. Therefore, the effects of approximately 1–35 ppt of sodium chloride were investigated. This result indicates that the electrostatic interactions between drug molecules and nanoparticles were screened with an increasing salt concentration. The results also confirm that the CRE using GME-CuNPs was sensitized by electrostatic force. Divalent salts are the main cause of water hardness that affects water quality and are important for fish culture. Importantly, Ca^2+^ and Mg^2+^ can form complexes with CIP [49]. This may affect the CRE of GME-CuNPs in CIP adsorption. In the present study, calcium salt solutions at concentrations of 0, 180, and 210 mg/mL were studied, as they represent low to high water hardness levels, respectively. The results indicate that calcium ions play an important role in the CRE of GME-CuNPs. Regarding Mg^2+^ effects, it is generally indicated by the WHO that magnesium ions are present in natural groundwater at low concentrations of approximately 50 mg/L [50]. However, previous work reported that the amount of magnesium salts in natural waters depends on the level of water hardness and can reach a maximum of 75 mg/L [51]. Our findings demonstrate that Mg^2+^ is also crucial for the CRE of GMS-CuNPs. As with Ca^2+^, a high concentration of Mg^2+^ significantly reduces the CRE. From these results, it is suggested that the elimination efficiency of GME-CuNPs can be a competition between the CIP cations and the divalent ions in the hardness water at the active sites of GME-CuNPs, resulting in the shielding of electrostatic attractions. Fish wastewater contains a variety of contaminants that are both environmental and fish-borne [48]. The water quality of the fish wastewaters during the present experiment was as follows: total ammonia nitrogen levels—0.1–0.2 ppm, and unionized ammonia—0.010–0.015 ppm. All wastewater samples had similar moderate amounts of total flavonoids, but the total phenolic contents were very different. The highest total phenolic contents were found in the wastewaters of *P. reticulate* and *O. niloticus*, respectively. Moreover, the flavonoid content of *P. reticulate* was higher than that of *O. niloticus*. In general, the flavonoid and phenolic functional groups can be adsorbed onto the surface of the nanoparticles to increase the negative charge of the particle surface and respectively increase the CRE of the nanoparticles. However, the CRE of the wastewater from *P. reticulate* was significantly less than that of *O. niloticus*. From these results, it is considered that the total phenolics, flavonoids, tannins, and ammonia concentrations in the fish wastewater did not exert a strong influence on the CRE of the GME-CuNPs. Considering the pH of all fish wastewaters, it is clearly seen that the pH of the *P. reticulata* wastewater was approximately 8.5–8.6, which was significantly higher than the others. The high pH of the *P. reticulata* wastewater may be due to the *P. reticulata* secretions, food scraps, microbial metabolites, and aquatic plants present in the water. It is considered that the elimination of CIP from water through the utilization of GME-CuNPs is a pH-dependent behavior. The pH range of 6–7, corresponding to the zwitterionic state of the CIP molecule, is the most suitable for obtaining the highest CRE value. In conditions with a pH above 7, the predominance of the negatively charged salt form of CIP at pKa_2_ (8.6) is evident [52,53], resulting in repulsion due to the negative charge of GME-CuNPs.

## 5. Conclusions

The obtained GME-CuNPs show high potential for CIP elimination with a CRE of 92.02 ± 0.09%. Both monovalent and divalent salts affected the CRE of the GME-CuNPs. A high salt concentration can significantly reduce the CRE. Contaminants in fish wastewater can decrease the CRE of GME-CuNPs. However, in the wastewaters of some fish species, GME-CuNPs have significant potential to achieve a CIP removal efficiency of more than 80%. In addition, the pH of the solution was also identified as an important factor influencing the CRE of the GME-CuNPs. These results suggest that adjusting the pH to a range of 6–7 in fish wastewater prior to treatment is necessary. These newly developed GME-CuNPs exhibited high performance in removing CIP from real fish wastewater samples. This points to the potential utility of GME-CuNPs in eliminating other antibiotics from aqueous solutions.

## Figures and Tables

**Table 1:** MS/MS fragmentation (negative and positive modes) of bioactive compounds in GME, and their tentative identification.

Peak No.	RT (min)	Detected *m*/*z*	Assigned Ion	MS/MS	Tentative Identification	Formula	Error (ppm)
1	2.303	185.0668	[M+H]^+^	127.0251, 84.9603	Pentane-1,2,2,3,3,4,4-heptol	C_5_H_12_O_7_	−6.60
2	3.145	118.0865	[M+NH_4_]^+^	97.0291, 59.0497	Isopropenyl acetate	C_5_H_8_O_2_	−2.07
3	3.285	209.0309	[M-H]^−^	191.0170, 129.0176, 85.0288, 59.0136	Glucaric acid	C_6_H_10_O_8_	−2.91
4	3.428	193.0722	[M+H]^+^	129.0552, 111.0447, 83.0496, 69.0340, 55.0544	Quinic acid	C_7_H_12_O_6_	−7.95
		191.0554	[M-H]^−^	127.0384, 85.0288, 59.0136			3.72
5	3.761	300.1258	[M+H]^+^	173.0454, 93.0553, 75.0445, 57.0341	Unidentified	-	-
6	3.989	133.0138	[M-H]^−^	115.0024, 71.0134	Malic acid	C_4_H_6_O_5_	3.36
7	4.546	189.0035	[M-H]^−^	127.0021, 96.9592, 83.0132, 57.0341	Oxalosuccinic acid	C_6_H_6_O_7_	3.05
8	4.770	222.0611	[M+NH_4_]^+^	169.0147, 113.0239, 97.0290, 58.0655	Daucic acid	C_7_H_8_O_7_	−1.22
9	4.863	191.0209	[M-H]^−^	111.0078.87.0084	Citric acid	C_6_H_8_O_7_	−6.15
10	4.906	236.0774	[M+NH_4_]^+^	201.0410, 183.0302, 155.0348, 109.0291, 81.0333, 59.0499	Ascorbic acid-6-acetate	C_8_H_10_O_7_	−3.90
		217.0347	[M-H]^−^	199.0220, 155.0326, 127.0388, 83.0496			3.12
11	7.097	166.0867	[M+H]^+^	120.0816, 103.0548, 77.0387	Phenyl alanine	C_9_H_11_NO_2_	−2.68
12	7.579	595.1456	[M+H]^+^	443.0991, 425.0889, 289.0719, 127.0394	Epicatechin-(4beta->8)- gallocatechin	C_30_H_26_O_13_	−1.65
		593.1317	[M-H]^−^	441.0767, 315.0464, 153.0181, 125.0227			
13	9.820	355.1042	[M+H]^+^	163.0402, 145.0297, 117.0330, 89.0389	Caffeoylquinic acid	C_16_H_18_O_9_	−5.19
		353.0875	[M-H]^−^	191.0541, 93.0333			0.87
14	10.103	579.1536	[M+H]^+^	427.1049, 409.0944, 333.0998, 291.0881, 247.0616, 163.0398, 127.0395	Procyanidin B2	C_30_H_26_O_12_	−6.73
		577.1356	[M-H]^−^	425.0830, 407.0724, 289.0690, 125.0242			−0.78
15	10.706	867.2184	[M+H]^+^	579.1529, 425.0882, 289.0724, 247.0612, 127.0396	Procyanidin trimer	C_45_H_38_O_18_	−6.12
		865.1987	[M-H]^−^	797.1475, 713.1401, 575.1106, 407.0715, 287.0526, 125.0223			−1.90
16	10.726	291.0879	[M+H]^+^	207.0667, 139.0399, 123.0448	Catechin	C_15_H_14_O_6_	−5.45
		289.0713	[M-H]^−^	245.0796, 151.0388, 125.0236, 57.0349			1.60
17	11.207	337.0931	[M-H]^−^	191.0544, 145.0282, 93.0341	Coumaroylquinic acid	C_16_H_18_O_8_	−0.62
		339.1102	[M+H]^+^	147.0452, 91.0541			−8.13
18	11.671	611.1634	[M+H]^+^	465.1035, 303.0517, 85.0288	Rutin	C_27_H_30_O_16_	−4.48
		609.1461	[M-H]^−^				0.01
19	11.862	741.1857	[M+H]^+^	571.1276, 451.1049, 289.0719, 179.0348, 123.0446	Cinchonain IIa	C_39_H_32_O_15_	−5.81
		739.1679	[M-H]^−^	587.1122, 289.0695, 125.0265			−1.43
20	11.990	433.1158	[M+H]^+^	313.0726, 283.0620	Pueraria glycoside	C_21_H_20_O_10_	−6.64
		431.0989	[M-H]^−^	311.0522, 217.0108, 151.0025, 59.0137			−1.23
21	12.798	451.1243	[M+H]^+^	305.0676, 191.0348, 129.0553, 85.0290	Astilbin	C_21_H_22_O_11_	−1.80
		449.1087	[M-H]^−^	303.0476, 285.0374, 151.0025, 65.0035			0.52
22	13.083	451.1252	[M+H]^+^	305.0677, 259.0614, 195.0300, 129.0555, 85.0290, 71.0497	Astilbin derivative	C_21_H_22_O_11_	−3.79
		449.1083	[M-H]^−^	303.0481, 285.0372, 151.0025			1.41
23	13.135	449.1083	[M-H]^−^	407.0721, 303.0478, 285.0377, 151.0025	Astilbin derivative	C_21_H_22_O_11_	1.41
24	13.321	373.2236	[M+H]^+^	211.1711, 193.1601, 175.1499, 135.1180, 109.1017, 69.0703	9-Hydroxy-7-megastigmen- 3-one glucoside	C_19_H_32_O_7_	−4.07
25	13.697	300.2552	[M+H]^+^	282.2446, 251.2010, 135.1167, 95.0860	N-Lauroyl Valine	C_17_H_33_NO_3_	−6.26
26	13.959	314.2712	[M+H]^+^	296.2604, 135.1181, 72.0814	Palmitoylglycine	C_18_H_35_NO_3_	−7.09
27	14.392	340.1049	[M+NH_4_]^+^	173.0459, 105.0342, 77.0383	Dulcisflavan	C_15_H_14_O_8_	−6.49
		321.0608	[M-H]^−^	297.0306, 199.0225, 155.0335, 127.0391			2.46
28	14.569	453.1204	[M+H]^+^	411.1099, 343.0837, 301.0729, 191.0355, 165.0563, 123.0450	Cinchonain Ia	C_24_H_20_O_9_	−5.28
		451.1032	[M-H]^−^	341.0627, 217.0118, 109.0289			0.57
29	17.257	274.2756	[M+H]^+^	230.2494, 149.0244, 88.0764, 57.0703	Unidentified	-	-
30	17.455	505.1385	[M-H]^−^	447.0674, 343.0748, 240.9990, 96.9593	Caryatin glucoside	C_24_H_26_O_12_	−6.63
31	32.277	403.2354	[M+H]^+^	259.1557, 217.0356, 185.0823, 157.0139, 129.0188, 57.0699	5S,15S-dihydroxy-9S,11R,8S,12S-diperoxy-6E,13E-eicosadienoic acid	C_20_H_34_O_8_	−6.83
32	32.714	379.1558	[M-H]^−^	324.0949, 281.0411, 180.9696, 112.9845	6-Deoxy-gamma-mangostin	C_23_H_24_O_5_	−1.85
33	33.817	395.1501	[M-H]^−^	351.0819, 283.0207, 152.9937, 78.9583	Gamma-mangostin	C_23_H_24_O_6_	−0.22

## Data Availability

The data are available upon request.
